# Genome-Wide ENU Mutagenesis in Combination with High Density SNP Analysis and Exome Sequencing Provides Rapid Identification of Novel Mouse Models of Developmental Disease

**DOI:** 10.1371/journal.pone.0055429

**Published:** 2013-03-01

**Authors:** Georgina Caruana, Peter G. Farlie, Adam H. Hart, Stefan Bagheri-Fam, Megan J. Wallace, Michael S. Dobbie, Christopher T. Gordon, Kerry A. Miller, Belinda Whittle, Helen E. Abud, Ruth M. Arkell, Timothy J. Cole, Vincent R. Harley, Ian M. Smyth, John F. Bertram

**Affiliations:** 1 Department of Anatomy and Developmental Biology, Monash University, Clayton, Melbourne, Australia; 2 Craniofacial Development Laboratory, Murdoch Children’s Research Institute, Royal Children’s Hospital, Parkville, Australia; 3 Molecular Genetics and Development Division, Prince Henry’s Institute of Medical Research, Clayton, Victoria, Australia; 4 The Ritchie Centre, Monash Institute of Medical Research and the Department of Obstetrics and Gynaecology, Monash University, Clayton, Melbourne, Australia; 5 The Australian Phenomics Facility, The Australian National University, Canberra Australian Capital Territory, Australia; 6 Research School of Biology, The Australian National University, Canberra, Australian Capital Territory, Australia; 7 Department of Biochemistry and Molecular Biology, Monash University, Clayton, Melbourne, Australia; Institut Jacques Monod, France

## Abstract

**Background:**

Mice harbouring gene mutations that cause phenotypic abnormalities during organogenesis are invaluable tools for linking gene function to normal development and human disorders. To generate mouse models harbouring novel alleles that are involved in organogenesis we conducted a phenotype-driven, genome-wide mutagenesis screen in mice using the mutagen *N*-ethyl-*N*-nitrosourea (ENU).

**Methodology/Principal Findings:**

ENU was injected into male C57BL/6 mice and the mutations transmitted through the germ-line. ENU-induced mutations were bred to homozygosity and G3 embryos screened at embryonic day (E) 13.5 and E18.5 for abnormalities in limb and craniofacial structures, skin, blood, vasculature, lungs, gut, kidneys, ureters and gonads. From 52 pedigrees screened 15 were detected with anomalies in one or more of the structures/organs screened. Using single nucleotide polymorphism (SNP)-based linkage analysis in conjunction with candidate gene or next-generation sequencing (NGS) we identified novel recessive alleles for *Fras1, Ift140* and *Lig1.*

**Conclusions/Significance:**

In this study we have generated mouse models in which the anomalies closely mimic those seen in human disorders. The association between novel mutant alleles and phenotypes will lead to a better understanding of gene function in normal development and establish how their dysfunction causes human anomalies and disease.

## Introduction

Genetic approaches in the mouse have been instrumental in aiding our understanding of the development, disease and congenital anomalies of the major organ systems. Most of our current knowledge has arisen through the use of “reverse genetic” or gene-driven approaches in which the gene of interest is mutated and the subsequent phenotype analysed. Although reverse genetics has provided us with a wealth of knowledge it has some limitations. It requires prior information about the function of the gene to be altered, is labour intensive, may result in an unexpected phenotype or no phenotype at all, and can only produce one mouse line at a time [1].

To circumvent some of these limitations, along with the recent advances in sequencing technologies, phenotype-driven (forward genetics) large-scale screens have come back in vogue using the chemical mutagen *N*-ethyl-*N*-nitrosourea (ENU). ENU is efficient and potent, inducing ∼1 mutation per 1–2.7 megabase of the genome. It creates point mutations, in coding or splice site regions, in a relatively unbiased fashion. These mutations better reflect the types of mutations responsible for human disease. ENU can induce different point mutations within the same gene, thereby creating an allelic series, each able to reveal a different aspect of the protein’s function which would not be revealed by a null allele. ENU injected into male mice introduces mutations into the genome of all cells including the spermatogonial stem cells allowing the mutations to be transmitted through the germ-line. Thousands of genes can be mutated in an efficient manner and mice screened for phenotypes of interest. Once inheritance of a phenotype is established the causal gene can be identified using linkage analysis and sequencing approaches [2], [3], .

Until recently, the bottleneck of ENU mutagenesis has been the identification of the causal gene. However, recent technological advancements in mapping and sequencing have made identification of the mutant gene more rapid. Typically, gene identification has previously been reliant on outcrossing the affected male to a different inbred strain. The progeny are then backcrossed or intercrossed. The chromosomal region containing the causal gene can then be identified by linkage analysis. Linkage analysis technologies are now available in high throughput formats using mouse strain specific single nucleotide polymorphisms (SNPs). Once linkage to a chromosomal region is identified candidate gene sequencing can be carried out. If candidates are not evident then recent advancements in next-generation sequencing technologies have made sequencing the exons of hundreds of genes within a chromosomal region feasible [8], [9], [10]. Alternatively to chromosomal linkage analysis whole exome sequencing may be used to identify the causal gene and point mutation [7].

Many ENU screening strategies have been employed. These include region-based screens to identify mutations in a defined region of the genome [11], [12], [13], [14], [15], [16], [17], sensitized screens [18], [19], [20], [21] and genome-wide screens [22], [23], [24], [25], [26], [27]. Many genome-wide, phenotype based screens have been performed to identify both dominant and recessive ENU-induced mutations. These screens have been extremely successful in identifying a range of novel alleles important for embryonic development and disease. Given that many recessive mutations are lethal it is necessary to perform these screens embryonically. Genome-wide embryonic recessive screens have focussed on specific developmental time points and specific phenotypes of interest [28], [29], [30], [31], [32], [33], [34].

In the present study we performed an ENU mutagenesis screen in embryonic mice to identify recessive mutations in genes involved in organogenesis. We brought together a team of developmental biologists (ENU Organogenesis Consortium), each with expertise in the development of a specific mouse organ system. We screened G3 embryos at embryonic day (E) 13.5 and E18.5 with a focus on identifying phenotypic abnormalities in limb and craniofacial structures, skin, blood, vasculature, lungs, gut, kidneys, ureters and gonads. A hierarchical and systematic strategy was developed in order to screen each of these structures/organs in every G3 embryo. We identified 15 mutant mouse lines exhibiting abnormalities in one or more of the structures/organs screened for and identified novel mutant alleles for *Fras1, Ift140 and Lig1.*

## Results

**Figure 1:**
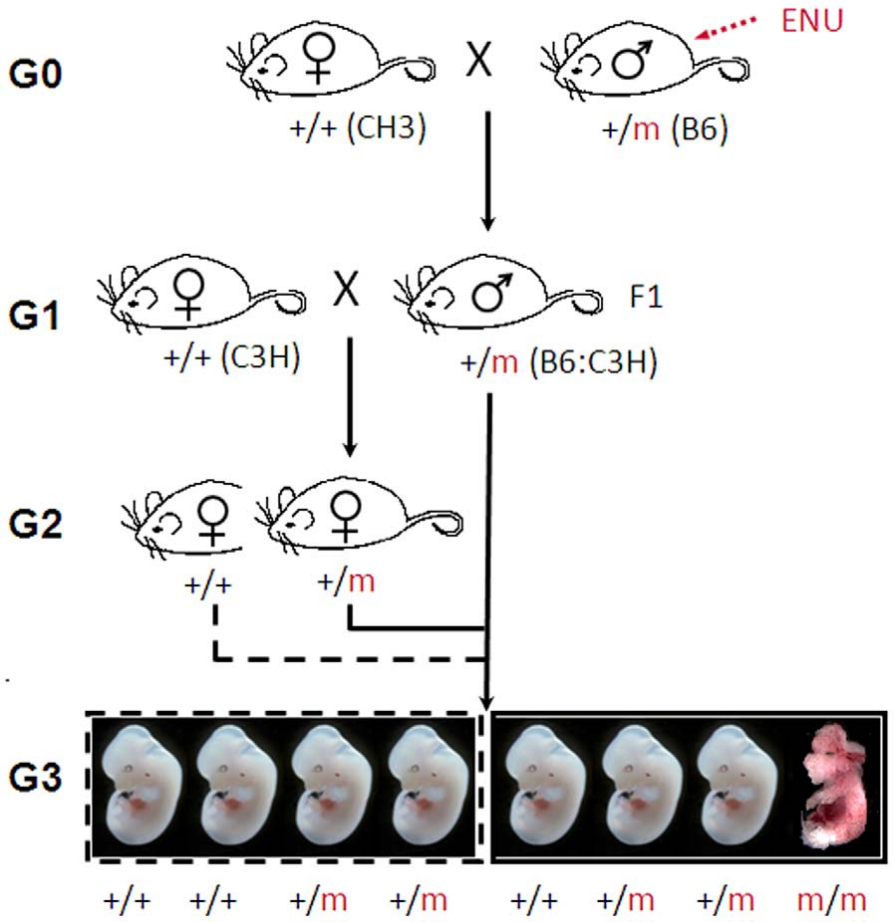
Breeding strategy for the generation of G_3_ embryos. C57BL/6J male mice were injected with ENU and bred with females of the inbred mapping strain, C3H/HeH (C3H). +/+ = wildtype, m = mutation, G = generation.

To screen for recessive mutations a standard three generation breeding protocol was used (Figure 1). Of the 52 pedigrees screened 25 were screened at E13.5 only, 14 were screened at E18.5 only and 13 were screened at both time points. We identified 15 pedigrees with a reproducible phenotype in multiple litters (Table 1). Embryos with a range of anomalies were identified including, structural anomalies (craniofacial, skeletal and limb defects, exencephaly, spina bifida and curled tail) and organ anomalies in the kidney, ureter, lung, skin, gonad, eye and blood. Line 12BCC-22 carried two mutations that resulted in the segregation of two pedigrees with distinct phenotypes (anaemia and spina bifida) (Table 1).

**Table 1:** Summary of ENU-induced mouse mutants.

Line	Time-point	Phenotype	Affected/Total no.embryos screened*	Affected/Total no. of embryos in affected litters	Gene ID/mapping
*bfb* #	18.5	skeletal (craniofacial/polydactyly)	16/149(11%)	9/46(20%)	*Fras1*
		skin		5/37 (3.5%)	
		kidney		2/21 (9.5%)	
11BC-11	18.5	Skeletal (syndactyly, craniofacial)	5/116 (4%)	5/20 (25%)	Unknown
snoopy#	13.5	craniofacial	8/134 (6%)	8/32 (25%)	Unknown
kanyon#	13.5	craniofacial, neural tube, exencephaly	12/52 (23%)	12/26 (46%)	Chr 7
*cauli* #	13.5	craniofacial, exencephaly, spina bifida, polydactyly, limb anomalies	15/73 (20.5%)	15/54 (28%)	*Ift140*
12BC-19	18.5	Kidney, ureter	12/208^b^ (6%)	12/64 (18.8%)^b^	unknown
12BC-20	18.5	Kidney, ureter	11/198^b^ (6%)	11/42 (26%)^b^	unknown
12BCC-13	18.5	lung	7/176 (4%)	7/21 (33%)	unknown^a^
12BCC-16	18.5	lung	3/27 (11%)	3/8 (37.5%)$	unknown^a^
12BCC-20	13.5	gonad	7/280^b^ (2.5%)	7/36 (19%)^b^	unknown^a^
12BCC22a#	13.5	blood	11/136 (8%)	11/29 (38%)	*Lig 1*
12BCC-22b	13.5	spina bifida, curled tail	23/136 (17%)	23/60 (38%)	unknown
12BCC-24	18.5	curled tail	4/24 (17%)	3/15 (20%)	unknown
		fused digit		1/15 (6.7%)	
14BC-2	18.5	eye pigmentation	3/31 (10%)	3/15 (20%)	Unknown
14BC-7	18.5	Kidney, ureter	7/305^b^ (2%)	7/43 (16%)^b^	Unknown

# Allele names and symbols have been registered with MGI. Allele ID for Ift140*^cauli^* is MGI: 4458412. Mouse strains have been deposited into the Australian Phenome Bank repository (http://pb.apf.edu.au/phenbank).

* Number of G3 embryos screened in litters with ≥6 embryos/litter.

$ Affected embryos in 3 individual litters. Litter sizes were <6 embryos for this pedigree.

a The phenotype was not inherited by the 2^nd^ generation males.

b Total numbers include male and female. Testis anomalies are only seen in males and kidney anomalies are predominately seen in males. Therefore a greater number of total embryos were screened.

### A Novel *Fras1* Allele Displaying Craniofacial, Skeletal, Skin and Urinary Tract Anomalies

**Figure 2:**
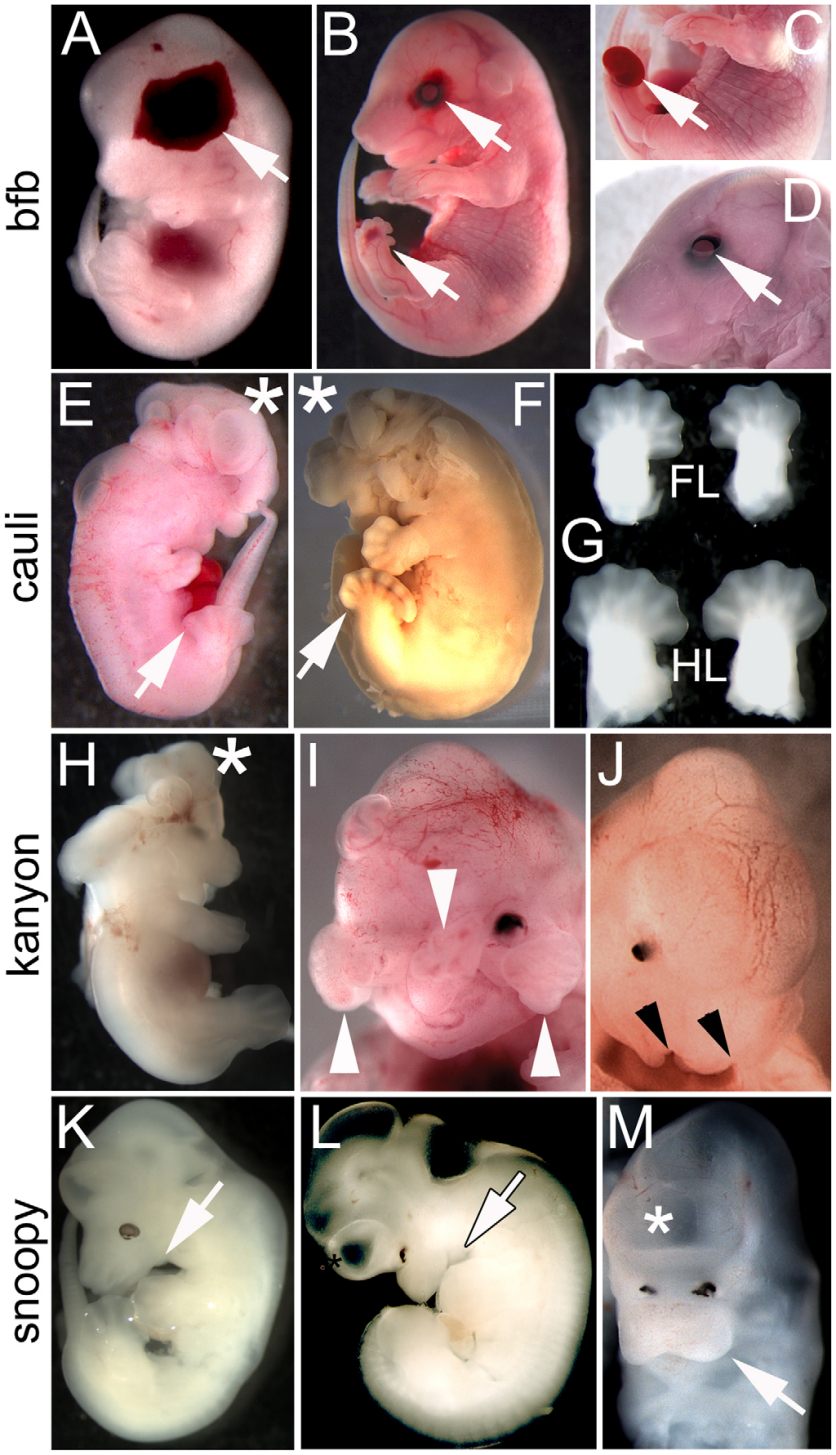
Craniofacial and skeletal mutants. (A–D) 11BC-5 (blood filled blisters; *bfb*), (E–G) 11BC-3 (*cauli*), (H–J) 12WT-49 (kanyon), (K–M) 12WT-9 (snoopy). *bfb* mutant at E13.5 (A) and E18.5 (B–D) exhibiting characteristic haemorrhagic blisters over the eye, side of the head and feet (arrows). The foot blisters can be discrete or distended as in (C) but are typically associated with digit malformations including polydactyly. The blisters over the eye are commonly associated with open eyelids (D). *Cauli* embryos at (E) E13.5 and (F) E16.5 present with exencephaly (asterisk) and polydactyly (arrow). (G) Fore- (FL) and hindlimbs (HL) of an E13.5 *cauli* embryo illustrating the variable autopod phenotype in the forelimbs. Kanyon embryos (H-J) frequently present with exencephaly (H, asterisk) and midfacial clefts. Clefts may result from a defect of frontonasal process development such that the maxillary and frontonasal processes (arrowheads) completely fail to fuse (I) or may present as bilateral cleft lip and palate (J) in mild cases. Regardless of the severity of the facial cleft, the eyes never develop normally (I, J). Snoopy embryos (K-M) present with forebrain malformation, poor eye development and mandibular hypoplasia/agnathia (arrow). (L, M) The forebrain often fails to divide into two vesicles (asterisk) and is associated with various degrees of hypotelorism (M).

**Figure 3:**
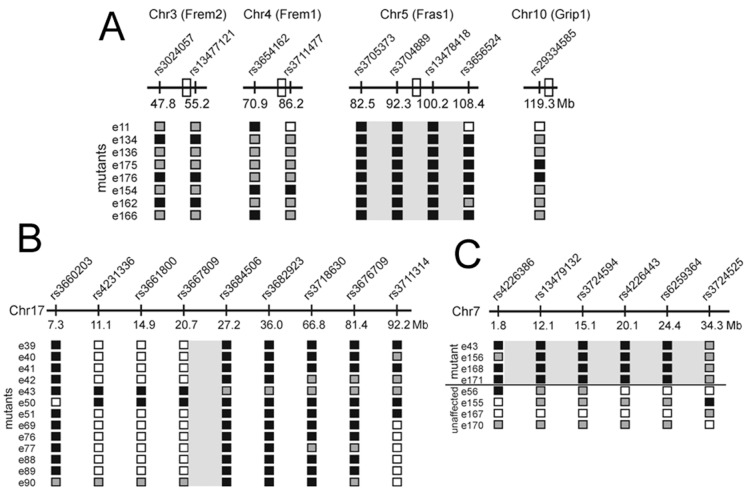
Linkage Analysis. (A) Based on the phenotype, a targeted approach was used to establish linkage to *Fras1* in the *bfb* strain. Polymorphic markers flanking the four previously characterised *blebs* genes (open boxes) were used to identify a candidate for sequencing. Strong linkage was observed with the *Fras1* gene (grey box). (B) A genome-wide scan established linkage to chromosome 17 in the *cauli* strain. Chromosome 17-specific markers were then used to refine linkage to a 7 Mb region between rs3667809 and rs3684506 (grey boxed region) containing 218 genes. No additional informative markers were available to further refine the linkage region. (C) A genome-wide scan established linkage to chromosome 7 in the 12BCC-22a line. Subsequent analysis using chromosome 7-specific markers refined linkage to a 32 Mb region containing approximately 700 genes. Numbers to the left in each diagram denote individual mutant embryos except in (C) where both mutant and unaffected embryos are shown. Black boxes denote homozygous C57BL/6 alleles, grey boxes denote C57BL6/C3H heterozygosity, white boxes denote untested markers.

Line 11BC-5 was originally identified as a strain that exhibited isolated cleft secondary palate at E18.5. Additional screening revealed a range of phenotypes including blood filled blisters on the head and feet at E13.5 and preaxial polydactyly, open eye lids (Figure 2A–D) and renal agenesis (data not shown) at E18.5. Based on the phenotype observed in E13.5 embryos, this strain was named blood filled blisters (*bfb*). This collection of phenotypic features is characteristic of the blebs mutants that arise following mutation of *Fras1*, *Frem 1, 2* and *Grip1* [35]. Targeted SNP analysis using markers polymorphic between C57BL/6 and C3H were used to investigate the possibility of linkage of the *bfb* phenotype to one of these four *blebs* genes using a cohort of 9 phenotypically mutant embryos. Markers flanking *Frem1*, *Frem2* and *Grip1* exhibited mixed genotypes (Figure 3A). In contrast, markers flanking *Fras1* were homozygous for the C57BL/6 allele in 9/9 samples, indicating clear linkage of the *bfb* phenotype to *Fras1*. Sequencing of all 75 exons and flanking sequences from genomic DNA of a single mutant identified a 10762T>C substitution (open reading frame of NCBI RefSeq transcript NM_175473.3) causing a Ser3588Pro mutation in FRAS1. PolyPhen-2 analysis predicts that this change is “probably damaging” and SIFT predicts that it is “not tolerated”.

### An *Ift140* Mutation Resulting in a Complex Ciliopathy

Line 11BC-3 was screened at E13.5 only and exhibits a very consistent phenotype involving exencephaly, anophthalmia, craniofacial malformation, hindlimb polydactyly and forelimb poly- and oligodactyly (Figure 2E–G). The hindlimbs were almost always bilaterally polydactylous while the forelimbs exhibited roughly equal proportions of oligodactyly, normodactyly and polydactyly which was uni- or bilateral (Figure 2G). In reference to the exencephalic morphology, this strain was named cauliflower (*cauli*). A significant proportion of E13.5 *cauli* embryos were in poor condition and the incidence of embryonic death increased rapidly with gestational age such that from 22 E17.5–18.5 litters only 7/137 (5.1%) *cauli* embryos have been recovered. The poor condition of many mid-gestation embryos made investigation of laterality defects impossible but a number of younger embryos have incidentally been observed to have heart looping defects (not shown). The impact of these early defects on later cardiac morphology is yet to be investigated. Conventional SNP-based linkage analysis identified a 7 Mb interval on chromosome 17 containing 218 genes (Figure 3 B). Given that the *cauli* mutant phenotype strongly resembled the phenotype of *Ift144* mutants [36], [37] a strong candidate gene within this interval was, *Ift140*, a component of the intraflagellar transport complex A (IFT-A) that regulates retrograde protein transport in ciliated cells [38], [39]. A 2564T>A substitution (open reading frame of NCBI RefSeq transcript NM_134126.3) resulting in an Ile855Lys mutation was identified in IFT140. PolyPhen-2 indicates that this change is “possibly damaging” while SIFT analysis predicts that it is “not tolerated”.

### Kanyon, a Model of Frontonasal Dysplasia

The 12WT-49 strain phenotype was identified at E13.5 and presented with defects in the frontonasal process, resulting in a mild to severe mid-facial cleft, exencephaly and neural tube defects (Figure 2 H–J). In severe cases the mid- and upper face was completely cleaved and this strain was therefore named kanyon. This phenotype is similar to a group of conditions known as frontonasal dysplasias (OMIM 136760) in which there are deficits in production of the mid-facial skeleton. Interestingly, the facial clefting and exencephaly while commonly observed together could also occur independently. There were no other gross phenotypes observable. Conventional SNP based mapping identified an approximately 67 Mb region on chromosome 7 between marker rs3713432 and rs4226997 containing approximately 1300 genes.

### A Mutant Displaying a Link between Holoprosencephaly and Mandibular Outgrowth

The 12WT-9 strain was identified at E13.5 and presented with forebrain anomalies, mandibular hypoplasia/aplasia, poorly developed eyes and hypotelorism (Figure 2 K–M). Given the combination of small eyes and lower jaw, this strain was named snoopy, in reference to its resemblance to the cartoon character of the same name. A small proportion of affected embryos presented with complete failure of forebrain vesicle separation and cyclopia. This suggests that the forebrain anomalies and hypotelorism seen in snoopy are part of a holoprosencephaly spectrum. The aetiology of the failed mandibular outgrowth is unclear and could be secondary to cranial neural tube defects. Mandibular hypoplasia/agnathia is associated with human holoprosencepahly in the condition agnathia-otocephaly complex (OMIM 202650). A whole exome sequencing approach is being used to identify the causative mutation in this strain.

### An Isoleucine to Phenylalanine Substitution in DNA Ligase 1 (Lig1) Results in Anaemia

During early mouse development, embryonic blood is first produced in the blood islands of the yolk sac (E8.5–E10.5), then later, during organogenesis (E11.5– E18.5), blood production switches to the fetal liver in order to meet the increasing needs of the circulatory and immune systems. At E13.5 haematopoietic cells constitute at least 50% of the fetal liver and most of these are haemoglobinised erythroid progenitors. Mutations that completely block erythropoiesis, such as *Gata-1* or *Fog-1* null mutations cause visible anaemia in the liver and circulation by E12.5 and are lethal between E12.5– E15.5 [40], [41].

**Figure 4:**
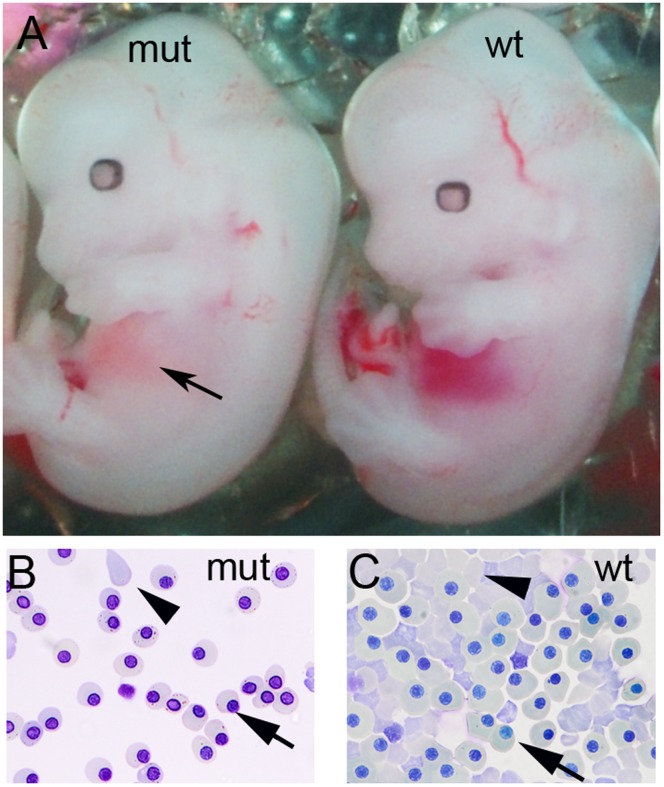
Anaemia in *Lig1* mutants. (A) A *Lig1* mutant (mut) with pale orange liver (arrow) compared to the red haemoglobin rich liver of a wildtype (wt) littermate. (B, C) Analysis of peripheral blood in E13.5 embryos by cytospin and Giemsa staining (x40). (B) *Lig1* mutant (mut) peripheral blood shows a high proportion of nucleated erythroblasts (arrow) to enucleated erythroblasts (arrowhead) compared with (C) a wild type (wt) littermate.

Based on these criteria we screened embryos for pale livers and identified E13.5 G_3_ embryos with visible anaemia in the liver in line 12BCC-22a (Figure 4A). Giemsa staining of peripheral blood verified that the embryos lacked mature enucleated red blood cells (RBC) (Figure 4 B, C). The gene responsible for this blood defect was localised to a 32.6 Mb region on the proximal end of chromosome 7 (rs4226386–rs3724525) (Figure 3 C). This region contained approximately 700 genes. Sequencing of all exons in this region using NGS identified a 1798A>T substitution in DNA ligase 1 *(Lig1)* (open reading frame of NCBI RefSeq transcript NM_001199310.1) resulting in an Ile600Phe mutation. The structure of Human LIG1 has been solved and we used this to predict that Ile 600 is on the surface that contacts DNA during DNA binding and repair [42]. This mutation alters the charge of this amino acid in the DNA binding and ATP dependant DNA ligase domain of LIG1. This may affect the ability of LIG1 to catalyse the joining of single strand DNA breaks during DNA replication, repair and recombination.

### Line 12BCC-20 Displays Testis Cord Abnormalities

**Figure 5:**
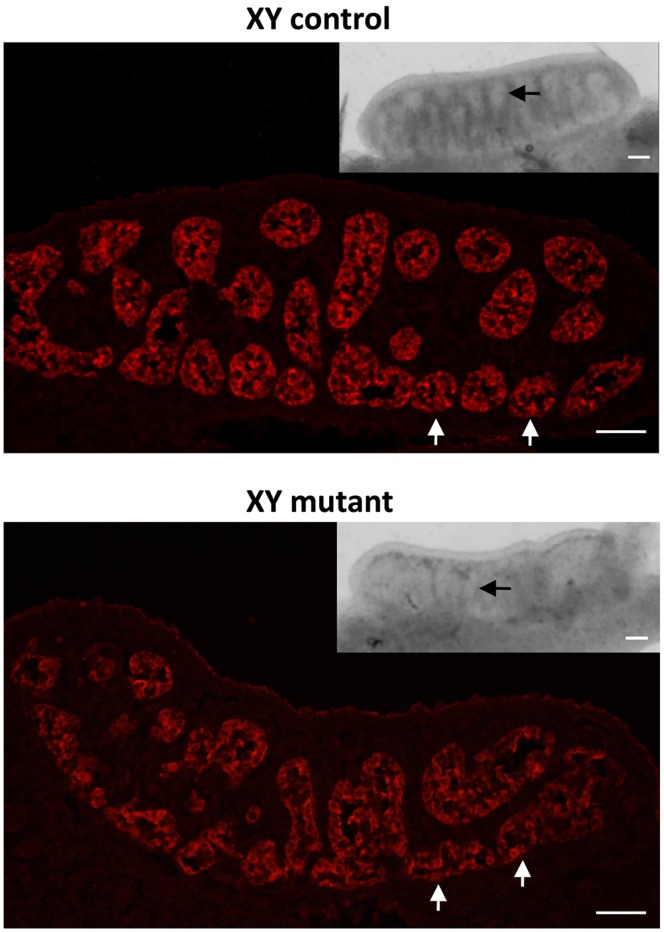
Testis cord defects in line 12BCC-20 at E13.5. Immunofluorescence staining for the Sertoli cell marker AMH (red, cytoplasm) on testis sections from unaffected (XY control) and affected (XY mutant) embryos. In affected embryos, testis cords are less organized when compared to unaffected embryos. **Insets:** Gross-examination of testes from unaffected and affected embryos. In affected embryos, testes are smaller and testis cords are barely detectable. Arrows indicate testis cords. All scale bars are 50 µm.

At E11.5, in the mouse, the bipotenital gonad commits to being either male (testis) or female (ovary) [43]. By E13.5, testes can be easily distinguished from ovaries by the presence of testis cords and their bigger size. In this screen we were interested in identifying mutants in which this decision had been disrupted resulting in an ovotestis phenotype. In addition, we were interested in mutations that may disrupt testis size, shape and testis cord formation. In line 12BCC-20, male G3 embryos with smaller testes were identified at E13.5 (7/36 embryos from affected litters (19.4%)) (Figure 5, insets). Moreover, while testis cords were clearly visible in unaffected gonads by gross examination, in affected gonads they were barely detectable (Figure 5, arrows in insets). Immunofluorescence staining for the Sertoli cell marker, AMH, revealed that in affected embryos the testis cords were less were less clearly defined when compared to unaffected embryos (arrows in Figure 5). The embryos with testis cord abnormalities were genotyped for chromosomal sex by PCR. All embryos had an XY karyotype; thus they did not display XX gonadal sex reversal. Unfortunately, the gonad anomalies were not detected in the second generation males possibly due to a weakly penetrant phenotype and the reduced contribution of the C57BL/6 mouse strain. C57BL/6 mice are exquisitely sensitive to testis development defects due to a higher gonadal expression of a female transcriptome relative to other mouse strains [44].

### Lines 12BC-19, 12BC-20 and 14BC-7 Display Kidney and Ureter Anomalies

Congenital anomalies of the kidney and urinary tract (CAKUT) commonly arise due to abnormal ureter development as a result of ectopic or lack of budding of the ureteric bud (presumptive ureter), ectopic ureter insertion into the bladder, abnormal smooth muscle cell or urothelium development and obstruction [45]. These defects, which are more commonly found in baby boys [46], [47], can lead to duplicated ureters, multiple kidneys, renal agenesis, hypoplasia and vesicoureteral reflux (backflow of urine into the kidney) causing ureter and renal pelvis dilation and hydronephrosis.

**Figure 6:**
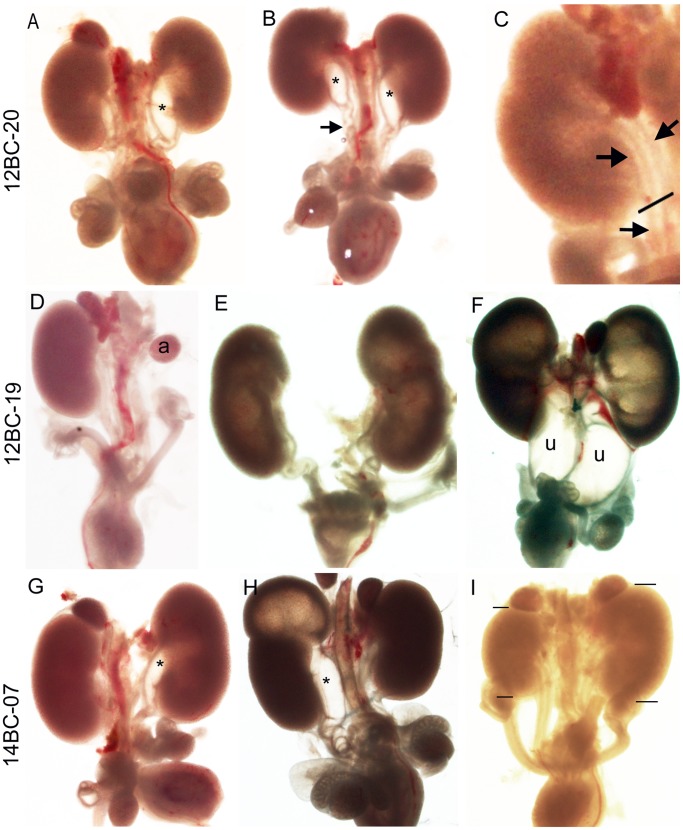
Urinary tract anomalies displayed by lines 12BC-19, 12BC-20 and 14BC-07. 12BC-20 (A–C) (A) Unilateral and (B) bilateral dilation of the renal pelvis (*). Kinks (A) and twisting (B) of the ureter (arrow) distal to the dilation. (C) Ureter bifurcation at the site marked with a line. Single ureter (arrow) below and bifurcated ureter (arrows) above the line. 12BC-19 (D–F) (D) Unilateral renal agenesis. (E) Bilateral hydronephrosis. (F) Bilateral hydroureter and hydronephrosis. 14BC-07 (G–I) (G) Dilated renal pelvis (*). (H) Duplicated ureter with hydroureter in one ureter leading to hydronephrosis in upper pole. (I) Unilateral renal hypoplasia (lines marking top and bottom of kidneys). a = adrenal, u = ureter.

Screening for these various kidney and ureter anomalies identified three affected lines, 12BC-20, 12BC-19, 14BC-7. In all lines we found a higher incidence of renal anomalies in males (82–67%) compared to females (18–33%). Kidneys from males in line 12BC-20 predominantly displayed dilation of the renal pelvis and proximal hydroureter either unilaterally (55%, 6/11 affected embryos) or bilaterally (36%, 4/11 affected embryos) (Figure 6 A–C). Several males had kinked ureters or severely twisted ureters distal to the dilation (36%, 4/11 affected embryos) (Figure 6 A and B). Females displayed either bifurcated ureters (9%, 1/11 affected embryos) (Figure 6 C) or two ureters with one entering the kidney and the other being blind-ended (9%, 1/11 affected embryos). In line 12BC-19 the majority of the affected embryos displayed dilation of the renal pelvis with or without hydroureter and hydronephrosis (58%, 7/12 affected embryos), 17% (2/12 affected embryos) displayed ureter duplication and 25% (3/12 affected embryos) displayed renal agenesis (Figure 6D–F). In line 14BC-7, 43% of the affected embryos displayed dilation of the renal pelvis (3/7 affected embryos), 43% displayed an alteration in kidney size or position which is unique to this pedigree (3/7 affected embryos), 7% displayed ureter duplication with hydroureter/hydronephrosis and 7% displayed unilateral renal agenesis (1/7 affected embryos) (Figure 6G–I). In all three lines the embryos did not exhibit any gross non-renal anomalies.

### Mutants with Lung Anomalies

Our primary focus for the lung screen was to identify mutants at E18.5 with thickened lung mesenchyme. The rationale being that during the late stages of fetal life there is a rapid increase in distal airspaces in the lung and a thinning of the mesenchyme between airspaces. These structural changes greatly enhance the gas exchange potential of the lung in preparation for birth when the lung must take on the role of gas exchange for the first time [48], [49]. If the lung mesenchyme fails to thin, it impairs gas exchange after birth and can be fatal in the absence of respiratory support [50].

**Figure 7:**
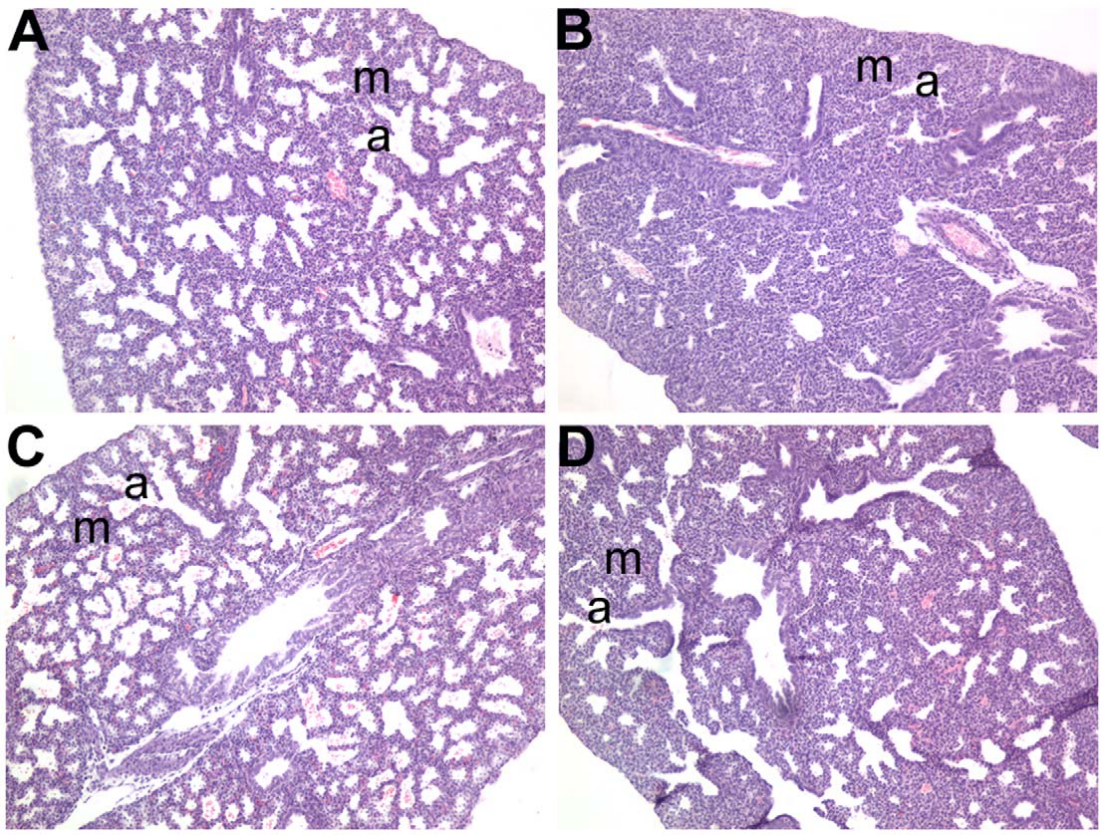
Lung anomalies displayed by lines 12BCC-013 and 12BCC-016. Lung histology in line 12BCC-013 (A, B) and 12BCC-016 (C, D). Affected lungs in lines 12BCC-013 (B) and 12BCC-016 (D) both displayed hypercellular, thickened lung mesenchyme (m) which can be clearly seen by the increased pink/purple staining of cells and their nuclei, between the small airways (a) in B and D compared to that in the lungs of unaffected littermate embryos (A and C), respectively.

To identify lungs with thick lung mesenchyme a histological screen was undertaken at E18.5 (just prior to birth). We identified two lines 12BCC-013 (7/21 embryos from affected litters, 33%) and 12BCC-016 (3/8 embryos from affected litters, 37.5%) with thick, hypercellular lung mesenchyme (Table 1, Figure 7). These mouse models offered the potential to increase our understanding of the molecular mechanisms that regulate thinning of the lung mesenchyme prior to birth. Unfortunately, the thick hypercellular phenotype was not detected in the second generation mice when searching for carrier males in line 12BCC-013. This may be due to a weakly penetrant lung phenotype or the introduction of a modifier allele that may block the phenotype. Line 12BCC-016 had to be abandoned as the G1 male mouse failed to produce further progeny.

## Discussion

The genetic insults responsible for many human congenital defects are yet to be discovered. Generating mouse models of congenital anomalies provides a valuable resource for understanding the aetiology and molecular mechanisms contributing to such congenital defects. Using ENU mutagenesis to generate such mouse models enables novel alleles to be associated with these conditions as well as providing valuable insights into the control of normal development. Our phenotype-driven, genome-wide ENU mutagenesis screen was designed to identify embryonic anomalies across multiple organs and structures within the same set of embryos. In addition, we successfully established a hierarchical and systematic protocol that could be used for future high throughput multi-organ screens. We generated ENU-induced mouse models that displayed craniofacial, skeletal, kidney, ureter, lung, skin, haematological and gonadal anomalies and have identified several novel alleles associated with some of these anomalies.

The most common anomalies identified in this screen involved structural defects (craniofacial, skeletal, limb and neural tube defects). Craniofacial anomalies comprise one third of all human birth defects and have a prevalence of 1% of live births worldwide [51]. Several pedigrees in this study displayed craniofacial anomalies. In particular, the kanyon and snoopy mutants exhibited phenotypes that mimic those seen in the human conditions, frontonasal dysplasias (OMIM 136760) and agnathia-otocephaly complex (OMIM 202650), respectively. Further characterisation of the kanyon and snoopy strains and the genetic mutation underpinning them will provide valuable information on the aetiology of similar conditions in humans.

Several mutants with defects in limb/digit formation were identified in this screen. Of particular interest was the *cauli* mutant which predominantly displayed exencephaly and polydactyly and is highly reminiscent of the phenotype seen in a number of sonic hedgehog (*Shh*) signalling pathway mutants [52], [53], [54]. The similarity of the *cauli* phenotype to existing *Shh* pathway mutant models provided very strong support for selecting *Ift140* as a candidate for sequencing. IFT140 is a core component of the retrograde intraflagella transport mechanism and is therefore a key component of the signalling nexus operating within the primary cilium [38], [39]. Further studies are required to determine the role Ift140 plays in limb/digit formation. Mutations in *IFT140* have recently been identified in Mainzer-Saldino syndrome (OMIM 266920) [55] further expanding the rapidly growing collection of human disorders caused by defects in primary cilia function.

The *bfb* mutant is an excellent example of a mouse model with multiple anomalies that were of interest to several investigators involved in this screen. The anomalies included blood filled blisters, isolated cleft palate, open eyelids, polydactyly and urinary tract defects. Fluid-filled or haemorrhagic blisters are the classic phenotype of the blebs mutant mice [35]. The first blebs mutant gene identified was *Fras1* [56], [57] and each of the subsequently identified blebs mutations are in genes encoding structurally or functionally related proteins [35]. Mutation of *FRAS1* in humans causes Fraser Syndrome which involves a series of related phenotypes to the blebs mutants [35]. The *Fras1* mutation identified in the *bfb* mutant occurs within a structurally uncharacterised domain between the CALXβ (calcium binding) domains and the transmembrane domain. The impact of this novel mutation on FRAS1 function is unclear but given the similarity of the *bfb* and *Fras1* null mice, it seems likely that the *bfb* mutation severely compromises FRAS1 function during early embryonic development.

The *Lig1* mutant displayed a heritable recessive anaemic phenotype characterised by a pale liver and reduced circulating blood. The anaemic phenotype of the homozygous *Lig1* mutants was associated with a relative decrease in mature RBCs and increased numbers of primitive, nucleated erythrocytes in circulating blood. This is consistent with the phenotype of the *Lig1* null mutant mice described by Bentley et al., (1996) [58]. Interestingly, a human compound heterozygote carrying two mutated *LIG1* alleles (Glu566Lys and Arg771Trp) has been described [59]. This patient displayed symptoms similar to Bloom syndrome, including immunodeficiency and increased cellular sensitivity to DNA-damaging agents, culminating in lethal lymphoma at age 19 [60]. Further analysis of our *Lig1* mutant may reveal the molecular role that this gene plays in erythropoiesis and carcinogenesis.

We identified several pedigrees with urinary tract abnormalities with the most common anomalies being dilation of the renal pelvis and/or ureter and hydronephrosis. Three of these mutants presented with non-syndromic renal and ureter anomalies and the *Fras1* mutant represented renal anomalies associated with a syndrome. These ENU mouse models are invaluable in studying the aetiology of the human condition CAKUT [61], which accounts for one-third of the anomalies detected by fetal ultrasound and can lead to end stage renal disease [62]. Whole exome sequencing is underway to identify the causal genes which will serve as candidate CAKUT genes for genetic screening of patients.

We have demonstrated that a multiple organ screen in embryonic mice performed remotely (The Australian Phenomics Facility, Canberra) to the multi-site investigators is highly feasible. In this screen, embryonic dissections were performed in a hierarchical and systematic manner (see Method S1), the technician was trained by the expert investigators to dissect and observe specific anomalies in the organs of interest. Phenotypes were recorded using digital photography and entered into the web-based, password protected database allowing all multi-site investigators to visualise phenotypes immediately. Organs and embryos were fixed and sent to the expert investigators for further analysis. The protocol we have described in detail (Method S1) forms the basis for establishing high throughput lethal screens in the future. The phenotyping protocol we outline in this report could be applicable to screening large numbers of embryonic lethal lines generated not only by ENU but also by gene targeting and gene trapping as is being undertaken by The International Knockout Mouse Consortium (IKMC). In many respects the throughput of our screen (more than 50 lines over a 2 year period) was limited not by the ability to undertake the phenotyping, but rather the generation of the ENU mutagenised pedigrees. We propose that the phenotyping protocol we describe would be easily applied to the analysis of 50–100 lines per year. Clearly this approach is not applicable to the average independent laboratory but our forecast throughput is well within the scope of existing and proposed high throughput lethal screens, including those encompassed and envisaged by the International Mouse Phenotyping Consortium (http://www.mousephenotype.org/) and the KOMP2 funding initiative (http://commonfund.nih.gov/KOMP2/). In addition, screening the same set of embryos for multiple organ anomalies provides a more comprehensive analysis of the mutant, allows the investigator to determine if they are dealing with a syndromic (as was the case with the *Fras1* mutant) or non-syndromic (isolated organ anomaly) condition, is more cost effective and facilitates teamwork and collaboration.

During the time in which this screen took place there were rapid advancements in new sequencing technologies. These new technologies have subsequently superseded the need to outcross mice to an inbred strain to perform recombination mapping. For the initial mutants identified in our screen, low-resolution genome mapping was performed using SNP genotyping to identify chromosomal linkage. In the case of line 12BCC-22a, linkage to an interval on Chr 7 was identified which contained over 700 genes. Next Generation Sequencing (NGS) eliminated the need to perform further breeding, which would have been required for recombination mapping and narrowing of the interval within which to select candidates for Sanger sequencing. NGS was used to sequence all exons in this interval leading to the identification of *Lig1* as the causal gene. For those mutants of interest, for which the causal genes are yet to be identified in this screen, chromosome specific or whole exome sequencing is being employed. Exome sequencing on average covers >95% of the consensus coding sequence (CCDS) exome [63]. Although mutations in non-coding regions are not isolated using exome sequencing, 99% of ENU mutations occur in splice sites and exons therefore exome sequencing should identify almost all ENU-induced mutations [7]. There will be some situations where mutations will be missed as a result of capture baits not covering specific genomic regions, the gene not being annotated as part of the CCDS, fragment mis-mapping or bad sequence coverage/quality due to high/low GC content [7], [64]. Despite these limitations gene mapping will be phased-out of future ENU-based screens and replaced with whole exome sequencing [7], [64]. Unlike recombination mapping, in which many affected and unaffected embryos are required to identify the mutated gene, exome sequencing initially only requires one affected embryo to identify the candidate casual mutation. ENU induced single nucleotide variations (SNVs) can then be subsequently validated in a larger cohort of embryos taking advantage of heritability information. This will rapidly reduce the time spent trying to identify the causative gene and allow mouse genotyping to be established earlier thereby reducing mouse husbandry costs. This will also undoubtedly decrease the percentage of valuable lines that are abandoned as a result of loss of phenotype during carrier searches and establishing heritability, as was the case in lines 12BCC-13 (lung anomalies) and 12BCC-20 (gonadal anomalies) in this study.

The efficiency of our screen at day E13.5 was 16% and at E18.5 was 33%. Many previous screens that have used ENU to identify genes that play a role during embryogenesis have focussed on earlier embryonic processes such as patterning and morphogenesis, often examining embryos at E9.5–10.5 [28], [65], [30], [66], [31], [32], or screening for post-natal lethality [11], [17], [67] and are therefore not directly comparable with our study. Additionally, some screens focused on mid-late gestation time-points but are not directly comparable because they used imaging techniques (rather than dissection) to reveal phenotypes [33], [68]. The recovery rate of mutations in our E13.5 screen was similar (but lower) to that of Ermakov et al [34] whom reported that 24% of pedigrees examined at E13.5 had consistent mutant phenotypes in two or more embryos, and our E18.5 screen was similar (but higher) to the 29% of phenotype containing pedigrees identified by dissection of E18.5 embryos by Herron et al [29]. Overall, the recovery rate reported in our study is similar to those previously reported in comparable screens.

Unfortunately, in our screen we did not recover a mutant displaying a gut phenotype. Our objective was to identify key regional gut patterning defects at E13.5 and gut epithelial defects at E18.5. The initial screening was based on identifying overall changes in morphology, length and diameter of the gastrointestinal tract in wholemounts. Although several embryos were highlighted for further examination after superficial examination, no overt anomalies were detected by histology. This indicates that attempting to screen for cellular defects based on overall morphology is not an effective method for identifying intestinal mutants. A more definitive method would be a screen based on histological criteria as was performed for the lung screen. Although feasible this is extremely labour intensive.

### Conclusions

In this study we have generated ENU-induced mutant mice that model human genetic disorders. We have identified novel mutant alleles of known genes which promise to provide further insight into the function of these genes. Despite several other ENU mutagenesis screens designed to identify recessive mutations that affect embryogenesis, the genes mutated in our screen have not been previously identified in these screens. This demonstrates the high potential for the discovery of novel alleles from such screens. The associations between phenotype and genotype which can be identified without prior gene bias, using ENU mutagenesis are invaluable in providing insight into the mechanisms that underlie normal development and human congenital disease.

## Methods

### ENU-mutagenesis

Animal experiments were conducted with approval of the Australian National University Animal Experimentation Ethics Committee and carried out in accordance with institutional guidelines. Male C57BL/6J mice were given three weekly intraperitoneal injections of 90 mg ENU/kg body weight as described previously [69]. After an 8 week recovery period the ENU treated male mice (G0) were crossed with either C57BL/6 or C3H/HeH (C3H) females to generate 89 founder males (F1). To screen for recessive mutations a standard three generation breeding protocol was used (Figure 1). F1 males which were heterozygous for the ENU induced mutations were crossed to C57BL/6J or C3H females to generate G2 progeny. The G2 daughters, half of which were heterozygous for the mutations, were backcrossed to their F1 fathers to generate G3 embryos that were screened at E13.5 and/or E18.5 for anomalies in skin, lung, kidney, ureter, vasculature, blood, gut, gonad and limb/craniofacial structures. Biopsies were taken from the embryos or yolk sacs for DNA extraction. A minimum of 4 litters were screened (average litter size 6–8 embryos and a minimum of 24 embryos) per pedigree and time-point. 12.5% of the G3 embryos were expected to be homozygous for the mutations. 52 lines were screened to completion with 37 additional lines abandoned due to breeding issues and small litter sizes.

### Screen

G3 embryos were examined for gross external visible anomalies of the skin, limb and craniofacial structures, haemorrhages, oedema (vascular abnormalities) and anaemia (blood abnormalities). Embryos were then dissected and lungs, gut, kidneys, ureters and gonads were microscopically examined. Blood, vascular and gonadal anomalies were examined microscopically at E13.5 only. Gonads were screened for ovotestes and abnormalities in testis cord formation (eg smaller gonads, fewer or disorganised cords). Lungs were examined histologically at E18.5 only. Skin, limb/craniofacial structures, kidneys, ureters and gut were examined at both time points. Anomalies were recorded using digital photography. Results and photographs were entered in a web-based, password-protected, in-house database, Musterer. All carcasses and organs were fixed in 10% formalin. A full description of the screening protocol is provided in the Supplementary Information (Method S1).

### Sex Genotyping of Mouse Embryos

Embryos were sexed by PCR using primers based on the mouse *Smcx* and *Smcy* genes as previously described [70]. Genomic DNA was isolated from the yolk sacs of embryos exhibiting testis anomalies for sex genotyping.

### Histology

All fixed E18.5 lungs were embedded in paraffin and 5 µm sections were stained with haematoxylin and eosin. Sections were examined for defects in distal lung development including condensed (thickened) lung mesenchyme, dilated distal airspaces, overt defects in vascular development and abnormal progression from pseudostratified columnar epithelial cells lining proximal airspaces to cuboidal/squamous epithelial cells lining the distal airspaces.

### Blood Analysis

Cytospins were performed on whole blood at 500×g for 4 minutes in a Cytospin 3 (Shandon). Cells were fixed in 100% methanol and stained with May-Grunwald-Giemsa.

### DNA Isolation

DNA was extracted from embryonic tissue using proteinase K digestion and precipitation. Approximately 50 mg of tissue was lysed at 55°C for 4 hours in 500 µl buffer containing 50 mM Tris.HCl, pH 8 (Sigma), 100 mM EDTA (Sigma), 0.5% SDS (Sigma) and 200 µg/ml proteinase K (Bioline). Following centrifugation at 18000×g for 10 min the supernatant was mixed with 150 µl 5 M NaCl (Sigma) and incubated at room temperature for 10 min. The supernatant from a second centrifugation (as previously) was mixed with 450 µl ice-cold isopropanol. The DNA was pelleted by a third centrifugation step and washed twice in 100 µl 70% ethanol prior to drying and resuspending in 100 µl of Tris.HCl buffered water. The quality and concentration of the DNA was determined by spectrophotometer absorbance readings at 260, 280 and 230 nm, followed by agarose gel electrophoresis analysis.

### Mapping

For linkage analysis genomic DNA from approximately 10–20 affected and 10–20 unaffected G3 C57BL/6J×C3H mice were screened with 41 strain specific SNP markers at proximal, distal and central positions on each chromosome using the Amplifluor SNP genotyping system (Chemicon, Millipore). Additional SNP markers were used within linked regions to further fine map the causal mutation in each strain.

### Sequencing

Sequencing of candidate genes was performed to locate the causal base substitution. DNA was prepared from an individual affected mouse. Primers were designed for candidate genes to amplify all exons +/−15 bp to cover splice junctions. Amplicons were then Sanger sequenced on an Applied Biosystems 3730*xl* capillary sequencer. This automated platform uses Big Dye Terminator (BDT) chemistry version 3.1 (Applied Biosystems). The raw trace files were analysed using Lasergene software (DNAstar) against the C57BL/6J mouse reference genome (mm9/NCBI m37).

### Next Generation Sequencing

A SureSelect custom solution array (Agilent) was designed using the online tool, eArray to include Refseq release 43 (NCBI) annotated exons (plus splice donor and acceptor sites) within the linkage interval. 100 bp paired end Illumina libraries of the captured regions from a single affected mouse were produced and run in a single lane of an Illumina GAIIx. Sequence reads were mapped to the NCBIM37 assembly of the reference mouse genome using the bowtie aligner47. Untrimmed reads were aligned allowing a maximum of two sequence mismatches and were discarded where they aligned to the genome more than once. Sequence variants were identified with SAMtools and custom perl scripts were subsequently used to identify those which occurred within exons and splice donor/acceptor sites, and were not known or strain-specific variants.

### Mutation Validation

Single nucleotide variations (SNVs) identified by Sanger or Next Generation Sequencing were validated using the Amplifluor SNP genotyping system (Chemicon, Millipore). Assays were designed to each SNV of interest and validated against a set of embryos affected for the phenotype of interest and a set of unaffected embryos. Amplifluor validation primers for *Fras1, Ift140* and *Lig1* are listed in Table S1. For the *bfb* line 39 embryos (26 unaffected and 13 affected), for the *cauli* line 37 embryos (29 unaffected and 8 affected) and for the *Lig1* line 20 embryos (10 affected and 10 unaffected) were genotyped to confirm the association of genotype to phenotype. In all cases the genotype correlated with the phenotype.

### Immunofluorescence

Testes were fixed, processed and sectioned as described previously [71]. For indirect immunofluorescence staining, frozen sections were incubated with Anti-AMH goat polyclonal antibody (1∶200; Santa Cruz, sc-6886) followed by detection with an Alexa dye-linked secondary antibody (1∶1000; Molecular Probes). Images were captured using fluorescence microscopy (Olympus Corp., NY, USA).

## Supporting Information

Table S1 **Amplifluor Genotyping Primers.**
(DOC)

Method S1 **ENU Organogenesis Screening Protocol.**
(DOC)
