# Supplementary material for: Genome-Wide ENU Mutagenesis in Combination with High Density SNP Analysis and Exome Sequencing Provides Rapid Identification of Novel Mouse Models of Developmental Disease
Source: PLoS One. 2013 Mar 1;8(3):e55429. doi: 10.1371/journal.pone.0055429 (PMC3585849; doi:10.1371/journal.pone.0055429)
Supplement: Table S1 — Amplifluor Genotyping Primers. (DOC) [file pone.0055429.s001.doc]

**Table S1** Amplifluor Genotyping Primers

| **Line** | **Gene** | **allele 1 primer** | **allele 2 primer** | **reverse primer** |
| --- | --- | --- | --- | --- |
| 12BCC-22a | *Lig1* | GAAGGTGACCAAGTTCATGCTGGAAGGTGGAGAAGTGAAGA | GAAGGTCGGAGTCAACGGATTTGGAAGGTGGAGAAGTGAAGT | ATGCGGCTGATAATGTCAGGAT |
| *bfb* | *Fras1* | GAAGGTGACCAAGTTCATGCTGTGGAGTGCTCAGACATTTGATC | GAAGGTCGGAGTCAACGGATTTTGTGGAGTGCTCAGACATTTGATT | TCGCCAGAGTTGATGAGGA |
| *cauli* | *Ift140* | GAAGGTCGGAGTCAACGGATTCACCAGCATGCCCAGCTGTT | GAAGGTGACCAAGTTCATGCTTTCACCAGCATGCCCAGCTGTA | AGCCAGAACTGGAAGCCAGA |
