# Supplementary material for: Genome-Wide ENU Mutagenesis in Combination with High Density SNP Analysis and Exome Sequencing Provides Rapid Identification of Novel Mouse Models of Developmental Disease
Source: PLoS One. 2013 Mar 1;8(3):e55429. doi: 10.1371/journal.pone.0055429 (PMC3585849; doi:10.1371/journal.pone.0055429)
Supplement: Method S1 — ENU Organogenesis Screening Protocol. (DOC) [file pone.0055429.s002.doc]

**Method S1. ENU Organogenesis Screening Protocol**

**A. Sequence of screen at E13.5 Sequence of screen at E18.5**

**Blood Blood**

**Skin Skin**

**Skeletal Skeletal**

**Gut Gut**

**Gonad Kidney**

**Kidney Ureter**

**Ureter Gonad**

**Lung**

**B. Dissections**

**B.1. E13.5 Dissection**

1. Sacrifice mother by cervical dislocation.
2. Remove uteri and place into ice cold PBS. Dissect embryos ASAP after collection. Preferably within 10 minutes of sacrificing the mother. This will ensure that the blood remains oxygenated and inside the embryo and yolk sac.
3. Carefully cut open the uterus without damaging the yolk sacs of the embryos.
4. Record litter size and the number of resorptions.
5. Dissect the embryos, keeping the yolk sac and placenta intact into a dish filled with cold PBS.
6. Record yolk sac vascularisation and morphology. Record haemoglobinisation and quantity of blood in yolk sac vitelline artery/vein.
7. Remove yolk sac and cut umbilical cord. Record the amount and colour of blood.
8. Observe embryonic vascularisation and morphology. Look for anaemia or signs of hypoxia (e.g. tissue necrosis). Check for haemorrhage and oedema. Record blood/haemoglobin in major blood vessels and liver.
9. If abnormalities observed photograph and prepare embryos in the litter as described in Section C1.
10. Observe skin and screen and photograph as described in Section C2.
11. Observe embryo for any skeletal defects, screen and photograph as described in Section C3.
12. Collect yolk sac for sex PCR, in case a gonad phenotype is seen, and place in labelled well of 6 well plate containing PBS. Place in a pre-labelled tube (10% formalin @ room temperature) if gonad phenotype is seen and if there is no yolk sac phenotype. If blood/vascular phenotype collect 1 hind limb and tail for sex PCR biopsy instead of yolk sac. Sex PCR only to be performed when screening on C57BL/6 background. If biopsy of limb and tail taken, photograph embryos prior to removal for skeletal screen.
13. Collect liver biopsy for DNA extraction for mapping and place in pre-labelled tube on ice and transfer to -80ºC. Liver biopsy to be collected from all embryos, affected and unaffected. If there is a blood phenotype, collect tail and 1 hindlimb for mapping DNA extraction, instead of liver. If biopsy of limb and tail taken, photograph embryos prior to removal for skeletal screen.
14. Dissect out gut, screen and photograph as described in Section C4. Place in a labelled well of 6 well plate containing PBS until all embryos of the litter have been dissected.
15. Dissect out gonads, screen and photograph as described in Section C5. Place in a labelled well of 6 well plate containing PBS until all embryos of the litter have been dissected.
16. Screen for kidney/ureter defects *in situ* and then *ex situ* and photograph as described in Section C6. Place in a labelled well of 6 well plate containing PBS until all embryos of the litter have been dissected.
17. Place remaining carcass in a labelled well of 6 well plate containing PBS until all embryos of the litter have been dissected.
18. Refresh the PBS between dissections.
19. Repeat steps 4-17 for each embryo of the litter.
20. When all embryos of the litter have been screened, if at least one embryo is found to be abnormal then fix all carcasses from the litter in tube full of 10% formalin to send to craniofacial/limb investigator. If skin defects are also seen on the carcass, then skin and craniofacial/limb investigators to coordinate analysis. If no abnormal embryos are found in the whole litter then all the embryos from that litter can be discarded once they are completely screened.
21. Photograph any organ abnormalities against normal littermates. Fix tissue that has shown any abnormalities along with unaffected tissue from littermates in separate tubes containing 10% formalin and send to appropriate Investigators.

**B.2 E18.5 Dissection**

1. Sacrifice mother by cervical dislocation.
2. Gently poke each of the pups through the uterus to see if they move (indicating the pups are still alive).
3. Quickly remove the pups from the uterus by cutting along the length of each uterine horn (along the opposite side to the placentas), then cut the umbilical cord.
4. Immediately place pups in the ice-cold PBS.
5. Screen for blood/vascular abnormalities as described in Section C1 and photograph when appropriate.
6. Observe skin and screen and photograph as described in Section C2.
7. Observe embryo for any skeletal defects, screen and photograph as described in Section C3.
8. Decapitate embryo and place head in a well of 6 well plate in PBS.
9. Collect yolk sac for sex PCR, in case a gonad phenotype is seen, and place in labelled well of 6 well plate containing PBS. Place in tube (10% formalin at room temperature) if gonad phenotype is seen. This will not interfere with the blood/vasculature screen as we do not expect to see any yolk sac phenotypes at E18.5.
10. Place entomology pins through all four limbs, close to the body (so as not to damage limbs) and into the dissecting gel and another pin at the top of the neck, to hold the embryo still during the dissection.
11. Open abdominal wall and dissect out liver. Collect liver biopsy for DNA extraction for mapping and place in pre-labelled tube on ice to be placed at -80ºC. Liver biopsy to be collected from all embryos, affected and unaffected. If there is a blood phenotype, collect tail and one hind limb for DNA extraction. If the latter is performed photograph embryos prior to removal of limb and tail for skeletal screen.
12. Remove gut, screen and photograph as described in Section C4. Place gut into well containing head for that embryo.
13. Observe urinary tract *in situ* and screen and photograph as described in Section C6.
14. Observe reproductive organs *in situ* and screen and photograph as described in Section C5.
15. Remove entire urinary/reproductive tracts, observe *ex situ*, and screen for any abnormalities. Place into well containing head for that embryo. If any abnormalities with urinary/reproductive organs fix them for the whole litter.
16. Using very fine scissors (e.g. Westcott spring scissors – straight) cut through the sternum continuing through the skin to mid-neck.
17. Spread the ribs apart and perform visual observations on the lungs – photograph any major abnormalities (i.e. total or partial lung agenesis).
18. Cut through the trachea and gently through any blood vessels and nerves that connect the trachea and heart to the upper thorax. Use a transfer pipette to drop some fresh PBS around the lungs to wash away any blood that obscures your vision of the lungs.
19. Lift the lungs gently, by scooping beneath them and once the nerves etc. at the top of the thorax are cut the lungs/heart should lift out easily to the level of the diaphragm.
20. Cut through the descending aorta and oesophagus, which connect the heart/lungs to the diaphragm, and remove heart and lungs *en bloc* and place into well containing head for that embryo.
21. Place carcass into same well that contains the head for that embryo.
22. Refresh PBS between dissections.
23. Repeat steps 3-20 for each embryo of the litter.
24. When all embryos of the litter have been screened, fix head and carcass in the same tube in 10% formalin (full tube) to send to craniofacial/limb investigator. If skin defects are also seen on the carcass then skin and craniofacial/limb investigators to co-ordinate analysis.
25. Fix lungs in full tube of 10% formalin and send to lung investigator.
26. Photograph any organ abnormalities against normal littermates. Fix tissue that has shown any abnormalities along with unaffected tissue from littermates in separate tubes containing 10% formalin and send to appropriate investigators.

**C. Screens**

**C.1 Blood/Vascular Development at E13.5 and E18.5 Primary screen**

**Dissection**

Observe yolk sac and embryonic vascularisation and morphology. Check haemoglobinisation and quantity of blood in yolk sac and embryo.

Look for anaemia or signs of hypoxia including tissue necrosis. Check for haemorrhage and oedema. Assess blood/haemoglobin in major blood vessels and liver.

**Record keeping**

When blood/vascular abnormality detected:

1. Capture colour digital still images of whole embryo with yolk sac attached using stereo-microscope digital camera. Also capture digital close-up images of abnormal embryo as required. Ensure that corresponding images are taken for at least one normal littermate.
2. Record date, pedigree and parent information, number of embryos (normal/affected/resorbed), description of abnormal phenotype. Image file names and tissues collected.

**Specimen Collection**

When one or more abnormal embryos detected, collect specimens from all pups and resorptions where possible:

1. For E13.5, make midline incision to open abdominal and pleural cavities. For E18.5 embryos, sagitally bisect using a new razor blade.
2. Collect biopsy for mapping from embryos and resorptions where possible. Photograph embryos prior to limb and tail removal for skeletal screen.
3. Place individual embryos with their yolk sacs into 10% formalin (room temperature) in labelled screw cap tubes for shipping.

**C2.** **Skin Primary E13.5 and E18.5 screen**

Observe embryos at E13.5 and E18.5 for any signs of blistering, folding or tautness.

At E18.5 also look for defects in whisker eruption, eyelids closed/open.

**Record keeping**

Photograph as appropriate to visualise abnormalities, with corresponding photographs of normal littermates.

**Specimen Collection**

Where any E13.5 embryo within a single litter is found to have an abnormal skin phenotype, all embryos from that litter are to be placed into individual tubes, after taking liver, and numbered accordingly and fixed in 10% formalin for shipping. Where all E13.5 embryos within a single litter are found to be normal, then those embryos can be discarded. Note that all E18.5 embryos are shipped to craniofacial/limb investigator, who will notify skin investigator if an abnormal skin phenotype has been found.

**C3. Skeletal Primary Screen at E13.5 and E18.5**

**At E13.5**

**Observe embryos for following abnormalities**

Failure of facial prominences to fuse.

Asymmetry of the skull or facial structures.

Missing or small eyes.

Short or abnormally shaped snout.

Differences in the size or shape of the limbs on the left and right sides.

Digits of hand and footplate should be completely separated.

Limbs should be distinct and protruding from the body wall and much longer than wide.

**At E18.5**

In addition to above observe for any asymmetry.

Cleft palate (in order to see this bottom jaw needs to be removed).

The skull should be one smooth curve from the base of the snout to the back of the neck.

Differences in the number or position of toes on the left and right sides.

A distinct change in angle should be seen in the forelimbs at the elbow or in the hind limbs at the ankle.

**Record Keeping**

Photograph any abnormal embryos along side normal litter mates.

**Specimen Collection**

Where any E13.5 embryo within a single litter is found to have an abnormal skeletal or craniofacial phenotype, all embryos from that litter are to be placed into individual tubes and numbered accordingly and filled with 10% formalin for shipping.  Where all embryos within a single E13.5 litter are found to be normal, then those embryos can be discarded once they have been completely screened.

All E18.5 embryos, whether abnormal or not, to be placed into individual tubes and numbered accordingly and filled with 10% formalin for shipping.

**C4. Intestinal primary screen at E13.5 and E18.5**

**Possible E13.5 phenotypes**

Intestinal hypoplasia/hyperplasia (demonstrated by a smaller/larger intestinal tract)

Patterning defects

Rotation defects

**Possible E18.5 phenotypes**

Epithelial hypoplasia/hyperplasia (demonstrated by a smaller/larger intestinal tract)

Imperforate anus

Dilation of intestinal tract

Rotation defects

Patterning defects

**E13.5 Dissection**

1. Carefully dissect embryo from yolk sac. Note that the intestine herniates through the body wall at this stage so care must be taken not to damage the intestinal tract at the first stage of dissection. Carefully pinch off the umbilical cord with forceps (do not pull as the intestine may be damaged).

2. Carefully dissect the intestinal tract from the embryo and remove connective tissue.

3. Compare the size of the intestinal tract to a normal control. Check for the presence of stomach, small intestine, caecum and hind gut. Note any changes in the size and organisation of these structures. Examine the diameter of the intestinal tract.

**Record Keeping**

If any abnormalities are present, photograph the intestinal tract in wholemount.

**Specimen Collection**

Only fix specimens from litters containing embryos with suspected phenotype for further screening by histology. Fix the whole intestine from each embryo in the litter in individual tubes in 10% formalin and ship to investigator.

**E18.5 Dissection**

The intestine is contained fully within the body at this stage and is filled with waste matter.

1. Carefully dissect the intestinal tract from the embryo and remove connective tissue.

2. Check for the presence of major morphological structures (stomach, small intestine, caecum and colon). Compare the size and position of these structures and the overall size of the intestinal tract with wild-type.

3. Note if meconium (yellow coloured contents) is present in the intestine and whether it is present along the length of the intestine.

4. Carefully flush the intestine with PBS using a syringe to remove intestinal contents.

5. Carefully examine the intestine under a dissecting microscope and note if any regions of the intestine appear dilated (larger diameter) or abnormally small. The intestine may also appear to have a thinner lining (i.e. under the dissecting microscope with light underneath will appear more translucent.

Slice a small segment of small intestine and check for the presence of villi under the microscope.

Compare size, diameter of intestine with normal controls.

Check the distal end of the colon. A blind ended tube indicates imperforate anus.

**Record Keeping**

Photograph any changes in wholemount.

**Specimen Keeping**

If one or more intestines with suspected phenotype are seen in a litter, fix the intestines from the entire litter in 10% formalin, in a separate tube for each embryo. Send the samples to investigator for further screening by histology, once all litters for the pedigree have been screened.

**C5. Gonad E13.5 and E18.5 Primary Screen**

**E13.5 Dissection**

Dissect out gonads to observe sex.

Look for ovotestis (contains both testicular and ovarian tissue).

Look for testis size.

Look for gonad shape - is it thicker in the centre of the gonad?

Count the number of testis cords - can they be clearly seen? (Average testis cord number is 12).

Look for the presence of the coelomic blood vessel in males and the absence in females (ectopic presence in females is more interesting than absence in males, as in males the blood may not yet be flowing through).

**E18.5 Dissection**

Dissect out testes (check that ovaries are present).

Look for testicular size.

Look for the male specific coelomic blood vessel.

Look for the structure of testis cords: Are they spotted or parallel?

**Record Keeping**

Photograph any abnormalities.

**Specimen collection**

If gonad abnormalities are seen fix yolk sacs in individual tubes filled with 10% formalin.

At E13.5 if abnormalities seen, fix both gonads from each embryo in individual tubes in 4% PFA .

At E18.5 if abnormalities are seen, fix the whole urogenital tract and reproductive system (which will include testes/ovaries/oviducts/uterus/epididymis/vas deferens and kidneys/ureters/bladder) in 4% PFA .

**C6. Kidney and Ureter Primary Screen**

**Possible E13.5 phenotypes**

Renal agenesis

Renal hypoplasia/hyperplasia

Renal dysplasia (abnormal shape)

Duplicated ureters

Multiple kidneys

Fused kidneys

Abnormalities in the ascension of the kidney (i.e. not located in the right place)

Above abnormalities can be unilateral or bilateral

**Possible E18.5 phenotypes**

Same as above also

Hydroureter

Hydronephrosis (associated with hydroureter)

Kinks/twists in ureters

Shortened ureters

Cystic kidneys (fluid accumulation in areas other than the pelvic region - kidney looks like it has blisters)

Above abnormalities can be unilateral or bilateral

**E13.5 and E18.5 Dissection**

Remove all organs described above leaving the kidneys, ureters and bladder intact in the embryo. Observe for phenotypes listed above. Can dissect the kidneys, ureters and bladder out of the embryo to look more closely for phenotypes.

**Record Keeping**

If there are any abnormalities then photograph whilst still in the embryo.

When abnormalities are found remove kidneys and ureters still attached to the bladder. Photograph kidney-ureter-bladder.

If abnormalities are bilateral then photograph the kidney-ureter-bladder tissue next to a normal looking littermate so we can compare the sizes.

**Specimen Collection**

Fix the kidney-ureter-bladder tissue from all embryos in the litter if any affected, in tube filled with 10% formalin for shipping.

**C7. Lung Primary Screen E18.5 Only**

**Possible gross phenotypes are:**

Lung agenesis

Lobe agenesis (there should be 3 lobes in the right lung and 2 lobes in the left lung)

Obvious hypoplasia, hyperplasia or dysplasia

Cystic lungs (fluid accumulation in regions of the lung with the appearance of blisters)

**Dissection**

Before placing the uterus or pups in ice-cold PBS, gently poke the embryos through the uterus to see if they move, indicating that they were alive at the time the mother was sacrificed.

Place pins through all four limbs, close to the body and into the dissecting gel and another pin at the top of the neck, to hold the embryo still during the dissection.

Follow steps 16-20 Section B.2 E18.5 Dissection for removal of the heart and lung *en bloc*.

**Record Keeping**

Photograph any visual lung abnormalities alongside the heart and lungs from littermates and include a note to the investigators stating which pedigree and embryos are abnormal.

**Specimen Collection**

Fix the heart and lungs from every E18.5 litter in 10% formalin for shipping to lung investigator for the primary histological screen of all lung samples.
